# Effects of Dietary Protein Level and Rumen-Protected Methionine and Lysine on Growth Performance, Rumen Fermentation and Serum Indexes for Yaks

**DOI:** 10.3390/ani14121751

**Published:** 2024-06-10

**Authors:** Haibo Wang, Jianhui Fu, Xia Wu, Yadong Wang, Wenjie Li, Yanling Huang, Jincheng Zhong, Zhongli Peng

**Affiliations:** 1 Key Laboratory of Qinghai-Tibetan Plateau Animal Genetic Resource Reservation and Utilization, Ministry of Education, Southwest Minzu University, Chengdu 610041, China; wanghaibo@swun.edu.cn (H.W.); fujianhui2022@126.com (J.F.); 18085292157@163.com (X.W.); 2 Key Laboratory of Animal Science of State Ethnic Affairs Commission, Southwest Minzu University, Chengdu 610041, China; 3 College of Animal and Veterinary Sciences, Southwest Minzu University, Chengdu 610041, China; wyd271013@163.com (Y.W.); liwenjie1026@163.com (W.L.); swunylh@163.com (Y.H.)

**Keywords:** yak, rumen-protected methionine, rumen-protected lysine, dietary protein, growth performance, rumen fermentation, blood index

## Abstract

**Simple Summary:**

The yak (*Bos grunniens*) is a multi-tasking grazing ruminant species playing vital roles for Tibetan nomads living in the Hindu Kush-Himalayan region and the Qinghai-Tibetan Plateau. Yaks are subjected to undernutrition in traditional pasture management systems during long winters due to inadequate forage intakes. This results in animals losing 25.7~30.2% of their live weight. Large ruminant animals have high intakes of feed and water. The utilization of dietary nitrogen efficiency of yaks is only about 25.9%, and a large amount of nitrogen is excreted in their feces and urine. Changes in nutritional measurement could improve the utilization of dietary nutrients and regulate the synthesis of proteins in yaks. Methionine and lysine are the first two limiting amino acids in a corn–soybean-meal-based diet for ruminants; therefore, we added rumen-protected methionine and lysine to low- and high-protein diets and found that the low-protein diet supplemented with rumen-protected methionine and lysine is beneficial for rumen and body health, physiological response, and metabolic status in yaks.

**Abstract:**

This study investigated the effects of the dietary protein level and rumen-protected methionine and lysine (RPML) on the growth performance, rumen fermentation, and serum indexes of yaks. Thirty-six male yaks were randomly assigned to a two by three factorial experiment with two protein levels, 15.05% and 16.51%, and three RPML levels: 0% RPML; 0.05% RPMet and 0.15% RPLys; and 0.1% RPMet and 0.3% RPLys. The trial lasted for sixty days. The results showed that the low-protein diet increased the DMI and feed conversion ratio of yaks. The diet supplemented with RPML increased the activities of IGF1 and INS and nutrient digestibility. The high-protein diet decreased the rumen butyrate concentration and increased the rumen isovalerate concentration. The low-protein diet supplemented with RPML increased the rumen pH and the concentrations of total volatile fatty acids, butyrate and NH_3_-N; the high-protein diet supplemented with a high level of RPML decreased the rumen pH and the concentrations of isobutyrate, isovalerate, propionate and NH_3_-N. The low-protein diet supplemented with RPML increased the total antioxidant capacity and glutathione peroxidase activity, along with the concentrations of malondialdehyde and amino acids such as aspartic acid, lysine, cysteine, etc. In conclusion, a low-protein diet supplemented with RPML is beneficial for rumen and body health, physiological response, and metabolic status in yaks.

## 1. Introduction

The yak (*Bos grunniens*) is a versatile grazing ruminant species providing meat, milk, hides, transportation, and dung as fuel for Tibetan nomads living in the Hindu Kush-Himalayan region and the Qinghai-Tibetan Plateau [1]. Yaks are subjected to undernutrition in traditional pasture management systems during long winters due to inadequate forage intakes. This results in yaks losing 25.7~30.2% of their live weight [2]. Large ruminant animals have high intakes of feed and water. The efficiency of dietary nitrogen utilization of beef cattle is only about 15.9~19.0%, and although yaks have higher urea recycling and greater microbe capture of the recycled urea by the rumen than beef cattle, their dietary nitrogen efficiency still only is about 25.9%, and a large amount of nitrogen is excreted in the feces and urine [3,4,5]. In addition, the crude protein content of grass (dead matter) in the cold season is less than 5% [6]. Hence, changes in nutritional measurement could improve the utilization of dietary nutrients and regulate the synthesis of proteins in yaks.

In recent years, research on yak nutrition has mainly focused on the effects of dietary energy and the concentrate to rough ratio on animal performance [7,8,9]. There has been limited research examining the effect of other dietary nutrients in yak diets on animal performance in the growing phase. Supplementary dietary protein could increase intramuscular lipogenic gene expression and decrease lipolytic gene expression, and eventually increase yak intramuscular fat (IMF) accumulation [10]. Providing rumen-protected amino acids in the diet may improve dietary nitrogen utilization and reduce nitrogen content in feces based on past dairy cattle research [11]. Lysine (Lys) and methionine (Met) have been identified most frequently as the first-limiting essential amino acids in metabolizable proteins for ruminant animals [12,13,14]. Waggoner et al. (2009) found that diets supplemented with 14 g of rumen-protected Met reduced the serum urea nitrogen content in Angus crossbred steers [15]. Another study reported that the live weight gain for growing steers fed grass silage can be increased by feeding small quantities of rumen-protected Met (RPMet) and rumen-protected Lys (RPLys), which corrected an amino acid deficiency [16].

Therefore, this study was designed to investigate the effects of different dietary protein and rumen-protected amino acid levels on growth performance, nutrient digestion, rumen fermentation, and serum biochemical parameters. One goal from this study was to provide technical information for the application of feeding rumen-protected Met and Lys (RPML) for yaks.

## 2. Materials and Methods

The feeding trial was carried out at the farm of Xinqiao Agriculture and Animal Husbandry Development Co. Ltd. (Aba, Sichuan Province, China, 2600 m altitude). The temperature of the barn during the trial ranged from −4 °C to 17 °C. Thirty-six male yaks (4 years of age; 219.0 ± 19.7 kg body weight) were selected and randomly assigned to a 2 × 3 factorial experiment evaluating two crude protein levels and three RPML levels. The low and high protein levels were 15.05% and 16.51%, respectively. The three RPML levels were as follows: (1) 0% RPML; (2) 0.05% RPMet and 0.15% RPLys; and (3) 0.1% RPMet and 0.3% RPLys. As a consequence, there were six dietary treatments: a low-protein diet supplemented with 0% RPML (CON1), a low-protein diet supplemented with 0.05% RPMet and 0.15% RPLys (LRPML1), a low-protein diet supplemented with 0.1% RPMet and 0.3% RPLys (LRPML2), a high-protein diet supplemented with 0% RPML (CON2), a high-protein diet supplemented with 0.05% RPMet and 0.15% RPLys (HRPML1), and a high-protein diet supplemented with 0.1% RPMet and 0.3% RPLys (HRPML2), with six replicates for each treatment and one yak for each replicate. Rumen-protected amino acids (RPMet: DL-methionine purity > 50% and RPLys: L-lysine > 47.5%) were sourced from Kemin Industries Inc. (Zhuhai, China). Diets were formulated to meet the nutritional requirements of beef cattle [17]. The ingredients and nutrient composition of the experimental diets are listed in Table 1. Yaks were vaccinated and dewormed before starting the experiment. The trial lasted for sixty days followed by a fifteen-day adaptation. Yaks were individually fed indoors and fed the total mixed rations twice daily at 8:00 and 14:00, respectively. RPML were added as a top dressing to the total mixed rations twice a day. Water was provided 4 h after each feeding.

### 2.1. Samples Collection

Initial and final body weights were recorded to calculate the average daily gain (ADG) at the beginning and end of the experiment, respectively. Feed intake and orts were recorded daily to calculate the dry matter intake (DMI). Then, the feed conversion ratio (FCR) could be obtained as the ratio of DMI to ADG. Starting on day 57 of the experiment, feed and fecal samples were collected on three consecutive days to conduct a digestion trial. A 10% tartaric acid solution was mixed with 25% of the total daily fecal output, which was then stored at −20 °C for the later determination of apparent nutrient digestibility. 

At the end of the experiment, the tail vein blood was collected and clotted for 2 h, then centrifuged at 3000× *g* for 15 min to separate the serum before morning feeding. At 3 h after morning feeding, rumen fluid samples were collected using a flexible esophageal tube (Kelibo Co, Ltd., Wuhan, China), the first 50 mL of rumen fluid was discarded to minimize saliva contamination, and another 60 mL of rumen fluid was then collected, in which 2 mL of rumen fluid was added to strain preservation tubes that were stored in liquid nitrogen for later analysis of ruminal microbiomes. Rumen fluid pH was measured using a pH probe (PHS-10, Century Fangzhou Technology Co., Ltd., Chengdu, China), and the remaining rumen fluid was filtered through four layers of sterile cheesecloth, filled into a 15 mL centrifuge tube and stored at −20 °C for the analysis of NH_3_-N concentration. 

### 2.2. Rumen Fermentation Parameters

Rumen fluid was centrifuged at 3000× *g* for 10 min to extract the supernatant, then 1 mL of supernatant was mixed with 0.2 mL of metaphosphoric acid (25 g/100 mL). The ruminal VFA was detected using a gas chromatograph (Agilent 6890N, Santa Clara, CA, USA). The VFA was measured according to the method described by Wang et al. [18]. The internal standard correction quantitative method was used for the calculation, and the internal standard was 2-ethylbutyric acid (2EB). The specific operation parameters are as follows: the carrier gas was N_2_; the average linear velocity was 38 cm/s; and the column pressure was 11.3 psi. The injection volume was 0.6 µL. The initial temperature was 120 °C for 3 min, and then was increased at 10 °C/min to 180 °C for 1 min. The inlet temperature and detector temperature were 210 °C and 250 °C, respectively. Ammonia-N (NH_3_-N) concentrations were determined using an alkaline phenol hypochlorite method [19] using a spectrophotometer (UV-1700, Shimadzu Corporation, Kyoto, Japan). 

### 2.3. Chemical Analysis

Diets were sampled and ground weekly and composited monthly. Composite samples were kept frozen at −20 °C prior to analysis. Association of Official Analytical Chemists (AOAC) methods [20] were used to determine ash, CP, ether extract, Ca, and P concentrations in the diets and fecal samples. The Ankom method [21] was used to determine neutral detergent fiber (NDF) and acid detergent fiber (NDF) contents. Acid-insoluble ash (AIA) was used as an endogenous tracer to determine apparent nutrient digestibility values according to the procedure reported by Van Keulen and Young [22], using the formula depicted by Wang et al. [23], as follows:

Nutrient digestibility = [1 − (Ad × Nf)/(Af × Nd)] × 100

where Ad (g/kg) and Af (g/kg) represent the AIA of the diet and feces, respectively, and Nd (g/kg) and Nf (g/kg) represent the nutrient contents of the diet and feces, respectively.

### 2.4. Serum Biochemical Parameters

The serum biochemical parameters including glucose (GLU), triglycerides (TG), total cholesterol (CHO), total protein (TP), urea, aspartate aminotransferase (AST), alanine aminotransferase (ALT), and alkaline phosphatase (ALP) were analyzed using an automatic biochemical analyzer (TC6010L, Tecom, Nanchang, China) according to the instructions of the kits.

Serum glutathione peroxidase (GSH-Px), superoxide dismutase (SOD), total antioxidant capacity (T-AOC), and malondialdehyde (MDA) values were measured using the dithiodinitrobenzoic acid method, the xanthine oxidase method, the ABTS radical cation, and the thiobarbituric acid (TBA) method, respectively. Serum catalase (CAT) activity was assayed following the method described by Goth [24]. Serum insulin (INS) and insulin-like growth factor 1 (IGF1) were analyzed according to the instructions of ELISA kits (Nanjing Jiancheng Bioengineering Ins., Nanjing, China). 

Serum-free amino acids were determined using a S-433D automatic amino acid analyzer (SYKAM GmbH, Munich, Germany). In brief, 0.5 mL of serum was mixed with 1 mL of 8% sulfosalicylic acid, centrifuged at 12,000 r/min for 10 min to precipitate proteins. Then, 0.2 mL of the supernatant was dissolved in 1.8 mL of the diluent solution and drawn and filtered using a 0.22 μm filter membrane, and then was detected using an amino acid analyzer. 

### 2.5. Statistical Analysis

All data were analyzed by the proc GLM of SAS 9.2 (SAS Inst. Inc., Cary, NC, USA) using the following model: Yij = μ + αi + βj + (αβ)ij + εij, where Yij is the dependent variable, μ is the population mean; αi is the fixed effect of the dietary protein level (i = 1 or 2), βj is the fixed effect of the rumen-protected amino acid level (j = 1, 2 or 3), (αβ)ij is the interaction between dietary protein level and amino acids level, and εij is the random error. In the event of a dietary protein–amino acid interaction, it was assessed post hoc using the Tukey test. *p* < 0.05 was considered as significant, and 0.05 < *p* < 0.10 was discussed as a tendency. 

## 3. Results

Initial and final BW were not affected by dietary protein, nor by RPML supplementation levels (*p* > 0.05) (Table 2). Yaks fed the high-protein diet tended to have higher ADG (650.0 g/d) than those fed the low-protein diet (831.6 g/d) (*p* = 0.090). The DMI of CON1 and HRPML1 were higher than those of CON2 and HRPML2, respectively (*p* < 0.05). The FCR of CON1 was higher than that of CON2 (*p* < 0.05); for a given dietary protein level, FCR was not affected when the RPML was supplemented into the diet (*p* > 0.05). RPML supplementation increased the activities of IGF-1 (*p* < 0.05) and INS (*p* < 0.001). 

As shown in Table 3, yaks fed the high-protein diet had higher NDF digestibility (40.42%) than those fed the low-protein diet (45.23%) (*p* < 0.01). The digestibility of NDF of HRPML2 was lower than that of CON2 (*p* < 0.05). The low-protein diet supplemented with RPML increased the digestibility of DM (*p* < 0.05). Dietary protein–RPML level interactions were present for OM, CP, and ADF digestibility values (*p* < 0.01). Organic matter, CP, and ADF digestibility values increased when the low-protein diet was supplemented with RPML, while the respective trait digestibility values decreased when the high-protein diet was supplemented with HRPML2.

The effects of dietary protein and RPML supplementation levels on rumen fermentation are presented in Table 4. Dietary protein–RPML level interactions were present for rumen pH and NH_3_-N concentrations and propionate concentrations (*p* < 0.05). Rumen pH and NH_3_-N concentrations generally increased when the low-protein diet was supplemented with RPML, while the respective trait values decreased when the high-protein diet was supplemented with RPML. Propionate concentrations were not affected by RPML supplementation when yaks were fed the low-protein diet, while propionate concentrations decreased when HRPML2 was provided to yaks fed the high diet. Yaks fed the high-protein diet had a lower butyrate concentration (15.50 mmol/L) and a higher isovalerate concentration (4.80 mmol/L) than those fed the low-protein diet (18.71 and 4.17 mmol/L, respectively) (*p* < 0.05). The low-protein diet supplemented with RPML increased the total VFA and butyrate concentrations (*p* < 0.05), and the high-protein diet supplemented with a high RPML level decreased the isobutyrate and isovalerate concentrations (*p* < 0.05). 

Dietary protein and RPML supplementation levels did not affect the serum biochemical parameters of yaks (*p* > 0.16) (Table 5). While dietary protein and RPML supplementation levels did not affect SOD activities (*p* > 0.22), dietary protein–RPML level interactions were present for MDA and GSH-Px activities (*p* < 0.03) (Table 6). MDA and GSH-Px activities increased when the low-protein diet was supplemented with RPML, while there were no differences in MDA activity when the high-protein diet was supplemented with RPML. CAT and T-AOC activities decreased (*p* ≤ 0.02) when the high-protein diet was fed to yaks versus feeding with the low-protein diet (CAT: 2.51 versus 2.10 U/mL, respectively, for low and high-protein diets; T-AOC: 4.13 versus 2.92 U/mL, respectively for low and high-protein diets), and CAT (*p* = 0.057) and T-AOC (*p* < 0.01) activities increased with RPML supplementation.

The effects of RPML and dietary protein level on serum-free amino acids are presented in Table 7. Dietary protein–RPML level interactions were present for essential amino acids, including tryptophan, valine, lysine, methionine, and nonessential amino acids, including glycine, alanine, cysteine, arginine and proline (*p* < 0.05). RPML supplementation increased the concentrations of tryptophan, alanine, arginine and proline when yaks were fed the low-protein diet, while there were no effects on the concentrations of tryptophan, alanine, arginine and proline when RPML supplementation was provided to yaks fed the high-protein diet. Use of a low level of RPML supplementation increased the concentration of valine when yaks were fed the low-protein diet to a greater extent than when a low level of RPML supplementation was used for yaks fed the high-protein diet. RPML supplementation increased concentrations of lysine and cysteine when yaks were fed the low-protein diet, while feeding only the high level of RPML increased lysine and cysteine concentrations when yaks were fed the high-protein diet. There was a greater increase in serum methionine concentrations with RPML supplementation of the high-protein diet as compared to RPML supplementation of the low-protein diet. RPML supplementation increased aspartic acid concentrations (*p* < 0.02) from 0.30% for no RPML supplementation to 0.39% for the low level of RPML supplementation and 0.57% for the high level of RPML supplementation.

## 4. Discussion

The feeding of rumen-protected amino acids has been shown to improve dietary nitrogen utilization and reduce fecal nitrogen content in dairy cattle [11]. In cattle, Lys and Met have been identified most frequently as the first-limiting essential amino acids in metabolizable proteins [12,13,14]. IGF-1 binds to insulin-like growth factor binding protein-3 to influence the growth, development, and reproduction in animals [25]. In the present study, although diets supplemented with RPML did not affect DMI and ADG; there was a significant increase in IGF-1 and INS concentrations. IGF-1 plays a role in inhibiting of protein degradation and improving protein synthesis and growth performance of animals [26]. INS could increase amino acid transportation and protein synthesis with a concomitant decrease in muscle protein breakdown, further increase muscle energy requirements [27]. In addition, INS promotes the growth of selected tissues by a direct action; in other tissues such as the musculoskeletal system, the action is indirect via the regulation of IGF release [28]. These indicate that RPML supplementation can affect yak growth performance. Consistent with our results, Zou et al. found that RPML supplementation increased serum IGF-1 for Holstein bulls [29]. In the present study, yaks fed with high-protein diet tended to have higher ADG, lower DMI and FCR than those fed with the low-protein diet, this indicate that feeding the high-protein diets had higher efficiency. 

In this study, yaks fed the high-protein diet with(without) RPML supplementation had higher NDF digestibility values than those fed with the low-protein diet. The reason may be that feeding the high-protein diet and RPML could better meet the rumen degradable protein requirements, such as amino acids, ammonia, and peptides of rumen microbes for maximum microbial protein synthesis [30]. Similarly to our results, Lee et al. found that total-tract NDF digestibility was decreased by the metabolizable protein deficient diets [31]. Protein available to ruminants is supplied by both microbial and dietary sources. Metabolizable protein is the true protein which is absorbed by the intestine and supplied by both microbial protein and protein which escapes degradation in the rumen; the protein which is available to the animal for maintenance, growth, fetal growth during gestation, and milk production [30]. In the present study, diet supplementation with RPML increased OM, ADF and CP digestibility values in yaks fed the low-protein diet, while OM digestibility values decreased in yak fed the high-protein diet supplemented with the high level of RPML. Previous study has shown that total-tract nutrients digestibilities could be suppressed with metabolizable protein deficient diets [32]. This would indicate that the basic diet needed to be supplemented with the limiting amino acids, Met and Lys to meet the amino acid requirements for maximum microbial protein synthesis in yaks.

Rumen pH is a key factor for the function of the rumen as it has profound effects on the microbial population and fermentation products as well as on the physiological functions of the rumen, primarily motility and absorption functions [33]. Rumen fermentation is relatively stable usually at a range of 5.8 to 6.5 in grain-adapted cattle [33]. In this study, high-protein diet significantly reduced rumen pH in yaks. An earlier study has shown that increasing dietary protein levels significantly increased rumen pH in cattle [34], another study found that dietary protein levels had no significant effect on rumen pH in lambs [35]; the differences on the effect of dietary protein level on rumen pH may be due to species differences and protein levels fed. In this study, low-protein diet supplemented with RPML increased the rumen pH, while high-protein diet supplemented with RPML decreased rumen pH. The reason may be that some of RPMet and RPLys in the rumen could be degraded, further be fermented by the microbiome and decrease the rumen pH, especially in the high-protein diet. In this study, feeding the high-protein diet decreased the butyrate concentration of rumen fluid compared to feeding the low-protein diet; this result is consistent with the low pH when yaks fed the high-protein diet. This result may be explained that fermentation promoting butyric acid production may produce less acid compared with fermentation promoting acetic and propionic acids [36]. Isovalerate is one of the branched chain VFA that is mainly derived from ruminal protein fermentation; ruminal concentrations of this branched chain VFA are used as a quick indicators of protein fermentation [37]. In this study, feeding the high-protein diet supplemented with the high level of RPML decreased the concentrations of the branched chain VFA, isobutyrate and isovalerate. Similar to our results, RPML supplementation of a metabolizable protein adequate diet tended to decrease isobutyrate concentrations in the rumen of lactating dairy cows [38]. Ammonia nitrogen is produced by the deamination and fermentation of peptides released during ruminal protein digestion [39]. In this study, RPML supplementation increased NH_3_-N concentrations in yaks fed the low-protein diet, while RPML supplementation decreased NH_3_-N concentrations in yaks fed the high-protein diet, which may indicate the low-protein diet could not met the yaks’ protein requirements and RPML supplementation may be a way to solve this issue. In the present study, RPML supplementation increased the concentrations of total VFA and butyrate; it was observed that acetate, butyrate, and NH_3_-N were main end-products of Lys [40], indicating that part of RPML were released into rumen fluid. In this study, dietary supplementation with high level of RPML decreased concentrations of isobutyrate and isovalerate for yaks fed the high-protein diet which may indicate better utilization of amino acids for protein synthesis in the animal. RPML supplementation did not affect propionate concentrations when yaks were fed the low-protein diet, while decreased propionate concentrations when yaks were fed the high-protein diet. This may be explained that when the dietary protein levels are relatively low, animo acids, such as Met or Lys may convert to propionate to satisfy the growth of yak. Similar to our results, researchers have found that when energy is limited, Met is ultimately converted to propionate by rumen bacteria and protozoa, and Lys can be converted to acetate and butyrate by rumen bacteria [40].

Blood urea nitrogen concentrations were previously found to have a positive relationship with dietary nitrogen requirements and expected protein digestibility [41]. In the present study, dietary protein and RPML supplementation levels did not affect serum biochemical parameters of yaks. MDA is a soluble degraded product of lipids [42] and an indicator of lipid peroxidation [43] that indicates oxidative damage in cells [44]. In this study, RPML supplementation increased MDA concentrations when yaks were fed the low-protein diet, while MDA concentrations were not affected when yaks were fed the high-protein diet. These results may indicate that excessive Met may cause lipid peroxidation and production of MDA [45]. In this study, RPML supplementation increased the activities of T-AOC, and tended to increase the activities of CAT. Besides, RPML supplementation increased GSH-Px activities when yaks were fed the low-protein diet, with no effect on GSH-Px activities when yaks were fed the high-protein diet. Among the RPML, Lys could be harvested by cells in large quantities, resulting in a metabolic reconfiguration that channels more NADPH into glutathione metabolism, reducing levels of reactive oxygen species and increasing tolerance to stress [46]. These results may indicate that RPML supplementation may increase antioxidant activities in yaks, and may be more beneficial when a low diet is fed. 

A dairy cow study found that diet supplementation with RPLys and RPMet could increase the content of Met and Lys in blood [47]. In the present study, RPML supplementation increased concentrations of tryptophan, glycine, alanine, arginine, proline, Lys and cysteine when yaks were fed the low-protein diet. Use of the low level of RPML supplementation increased the concentration of valine when yaks were fed the low-protein diet to a greater extent than when the low level of RPML supplementation was used for yaks fed the high-protein diet. The efficiency for utilization of dietary essential amino acids will increase with decreasing metabolizable protein supply [38]; hence the utilization of Met and Lys in the yaks fed the low-protein diet is higher. In addition, by providing RPML supplementation for the low-protein diet, the added Met and Lys could be used with amino acids absorbed from the gastrointestinal tract to better meet the amino acid requirements for tissue protein synthesis for yaks. Consistent with our results, Veria et al. found that feeding with RPLys and RPMet increased plasma free Lys concentrations [16].

## 5. Conclusions

In conclusion, feeding with a high-protein diet tended to increase ADG in yaks; RPML supplementation for the low-protein diet could improve AA supply in the diet to increase nutrient digestion, improve rumen fermentation and antioxidant capacity, and increase serum-free amino acids in yaks. RPML supplementation for the low-protein diets may be suitable for managing the diets of yaks.

## Figures and Tables

**Table 1:** Ingredients and chemical composition of the low- and high-protein diets.

Item	Low-Protein Diet	High-Protein Diet
Ingredient (%)		
Corn grain	40	36
Corn silage	25	25
Corn distillers	25	25
Soybean meal	7.7	11.65
Mineral-vitamin premix ^1^	1.15	1.15
Limestone	0.5	0.5
Salt	0.4	0.4
Urea	0.25	0.3
Nutrition level		
CP (%)	15.05	16.51
ME (MJ/kg)	12.31	12.29
OM (%)	94.27	94.04
EE (%)	3.81	3.73
NDF (%)	23.39	23.44
ADF (%)	12.18	12.33
Lysine (%)	0.4	0.49
Methionine (%)	0.22	0.24

^1^ Every kilogram of mineral–vitamin premix contained the following: vitamin A, 400,000 IU; vitamin D, 45,000 IU; Fe, 6 g; Zn, 4.2 g; Cu, 2.5 g; Mn, 10.25 g; I, 50 mg; Co, 20 mg; and Se, 54 mg. CP: crude protein; ME: metabolizable energy; OM: organic matter; EE: ether extract; NDF: neutral detergent fiber; ADF: acid detergent fiber.

**Table 2:** Effects of dietary protein level and amounts of rumen-protected lysine and methionine supplementation on the growth performance, insulin and insulin-like growth factor of yaks.

Item	Low Protein			High Protein			SEM	*p*-Value		
	CON1	LRPML1	HRPML1	CON2	LRPML2	HRPML2		CP	AA	CP × AA
Initial BW (kg)	219.8	219.3	219.5	218.8	218.4	218.3	3.28	0.677	0.694	0.423
Final BW (kg)	254.6	262.3	258.8	279	264.7	265.4	4.77	0.666	0.725	0.269
ADG (g/d)	579.2	716.7	654.2	938	770.8	786.1	37.81	0.09	0.352	0.217
DMI (kg/d)	6.15	6.11	6.1	6.04	6.03	5.99	0.016	0.001	0.179	0.875
FCR	11.23	8.63	9.6	7.7	7.83	7.77	0.468	0.03	0.496	0.428
IGF-1 (ng/mL)	105.4	118.8	112.3	97.6	123.9	114.7	3.088	0.981	0.024	0.537
INS (mIU/L)	6.19	9.9	10.4	5.73	9.66	7.85	0.511	0.037	<0.001	0.114

CON1: low-protein diet supplemented with no rumen-protected methionine and lysine; LRPML1: low-protein diet supplemented with 0.05% rumen-protected methionine and 0.15% rumen-protected lysine; LRPML2: low-protein diet supplemented with 0.1% rumen-protected methionine and 0.3% rumen-protected lysine; CON2: high-protein diet supplemented with no rumen-protected methionine and lysine; HRPML1: high-protein diet supplemented with 0.05% rumen-protected methionine and 0.15% rumen-protected lysine; HRPML2: high-protein diet supplemented with 0.1% rumen-protected methionine and 0.3% rumen-protected lysine; BW: body weight; ADG: average daily gain; DMI: dry matter intake; FCR: feed conversion ratio; efficiency; IGF-1: insulin-like growth factor 1; INS: insulin.

**Table 3:** Effects of dietary protein level and amounts of rumen-protected lysine and methionine supplementation on the nutrient digestibility of yaks (%).

Item	Low Protein			High Protein			SEM	*p*-Value		
	CON1	LRPML1	HRPML1	CON2	LRPML2	HRPML2		CP	AA	CP × AA
DM	53.49 ^a^	59.06 ^b^	56.68 ^a,b^	55.60 ^a,b^	55.98 ^a^	55.36 ^a^	0.533	0.378	0.038	0.067
OM	48.34 ^a^	54.79 ^c^	52.09 ^b^	52.73 ^b^	52.34 ^b^	49.23 ^a^	0.547	0.385	<0.001	<0.001
CP	52.20 ^b^	56.67 ^c,d^	58.13 ^d^	53.99 ^b,c^	52.57 ^b^	47.68 ^a^	0.871	<0.001	0.147	<0.001
NDF	38.39	43.18	39.7	47.04	46.95	41.71	0.949	0.002	0.038	0.107
ADF	55.69 ^a^	63.75 ^b,c^	62.99 ^b,c^	63.4 6 ^b,c^	66.31 ^c^	61.97 ^b^	0.875	0.005	0.001	0.006

^a–d^ Values within a row lacking a common superscript differ (*p* < 0.05) due to diet protein–rumen-protected methionine and lysine supplementation interaction. CON1: low-protein diet supplemented with no rumen-protected methionine and lysine; LRPML1: low-protein diet supplemented with 0.05% rumen-protected methionine and 0.15% rumen-protected lysine; LRPML2: low-protein diet supplemented with 0.1% rumen-protected methionine and 0.3% rumen-protected lysine; CON2: high-protein diet supplemented with no rumen-protected methionine and lysine; HRPML1: high-protein diet supplemented with 0.05% rumen-protected methionine and 0.15% rumen-protected lysine; HRPML2: high-protein diet supplemented with 0.1% rumen-protected methionine and 0.3% rumen-protected lysine; DM: dry matter; OM: organic matter; CP: crude protein; NDF: neutral detergent fiber; ADF: acid detergent fiber.

**Table 4:** Effects of dietary protein level and amounts of rumen-protected lysine and methionine supplementation on rumen fermentation of yaks.

Item	Low Protein			High Protein			SEM	*p*-Value		
	CON1	LRPML1	HRPML1	CON2	LRPML2	HRPML2		CP	AA	CP × AA
pH	6.63 ^c^	6.69 ^b,c^	6.75 ^d^	6.63 ^c^	6.52 ^b^	6.39 ^a^	0.036	<0.001	0.074	0.001
NH_3_-N (mg/dL)	9.95 ^a^	11.13 ^b^	12.95 ^c^	12.24 ^c^	9.88 ^a^	8.90 ^a^	0.435	0.006	0.2	<0.001
Total VFA (mmol/L)	72.6	116.76	152.79	102.73	101.08	124.69	6.61	0.696	0.005	0.115
Acetate (mmol/L)	58.28	57.84	54.62	55.33	60.21	58.26	0.618	0.373	0.151	0.059
Propionate (mmol/L)	15.16 ^a^	16.17 ^a,b^	14.67 ^a^	19.67 ^c^	18.16 ^b,c^	15.21 ^a^	0.434	<0.001	0.003	0.031
Isobutyrate (mmol/L)	2.94	2.59	2.36	3.01	2.95	2.48	0.068	0.112	0.001	0.523
Butyrate (mmol/L)	17.03	16.95	22.14	14.51	11.14	17.85	0.779	0.001	<0.001	0.459
Isovalerate (mmol/L)	4.29	4.22	4.01	5.12	5.2	4.07	0.142	0.015	0.048	0.261
Valerate (mmol/L)	2.32	2.24	2.21	2.37	2.34	2.14	0.042	0.765	0.292	0.754

^a–d^ Values within a row lacking a common superscript differ (*p* < 0.05) due to diet protein–rumen-protected methionine and lysine supplementation interactions. CON1: low-protein diet supplemented with no rumen-protected methionine and lysine; LRPML1: low-protein diet supplemented with 0.05% rumen-protected methionine and 0.15% rumen-protected lysine; LRPML2: low-protein diet supplemented with 0.1% rumen-protected methionine and 0.3% rumen-protected lysine; CON2: high-protein diet supplemented with no rumen-protected methionine and lysine; HRPML1: high-protein diet supplemented with 0.05% rumen-protected methionine and 0.15% rumen-protected lysine; HRPML2: high-protein diet supplemented with 0.1% rumen-protected methionine and 0.3% rumen-protected lysine; VFA: volatile fatty acid.

**Table 5:** Effects of dietary protein level and amounts of rumen-protected lysine and methionine supplementation on the serum biochemical parameters of yaks.

Item	Low Protein			High Protein			SEM	*p*-Value		
	CON1	LRPML1	HRPML1	CON2	LRPML2	HRPML2		CP	AA	CP × AA
GLU (mmol/L)	1.74	2.32	2.45	3.26	1.8	1.97	0.288	0.79	0.849	0.366
TG (mmol/L)	0.1	0.13	0.15	0.21	0.09	0.13	0.023	0.714	0.775	0.479
CHO (mmol/L)	0.54	0.95	0.83	0.83	0.79	0.76	0.053	0.848	0.405	0.261
TP (g/L)	22.15	30.25	25.4	36.9	25.47	25.13	2.586	0.581	0.828	0.375
ALB (g/L)	12.2	16.25	12.6	18.13	11.97	12.07	1.245	0.892	0.691	0.331
Urea (mmol/L)	1.95	2.14	2.06	3.23	2.14	2.77	0.184	0.081	0.573	0.349
LDH (U/L)	407.3	556.8	506.4	524.2	463.4	394.7	28.8	0.645	0.723	0.292
ALT (U/L)	12.75	17.75	17.6	18.4	15.53	14.97	1.077	0.915	0.936	0.336
ALP (U/L)	86.1	124.5	128.3	131.4	87.4	129.5	10.94	0.9	0.712	0.42
AST (U/L)	28.65	47	45.75	51.17	43.07	35.33	3.314	0.697	0.801	0.165

CON1: low-protein diet supplemented with no rumen-protected methionine and lysine; LRPML1: low-protein diet supplemented with 0.05% rumen-protected methionine and 0.15% rumen-protected lysine; LRPML2: low-protein diet supplemented with 0.1% rumen-protected methionine and 0.3% rumen-protected lysine; CON2: high-protein diet supplemented with no rumen-protected methionine and lysine; HRPML1: high-protein diet supplemented with 0.05% rumen-protected methionine and 0.15% rumen-protected lysine; HRPML2: high-protein diet supplemented with 0.1% rumen-protected methionine and 0.3% rumen-protected lysine; GLU: glucose; TG: triglycerides; CHO: cholesterol; TP: total protein; ALB: albumin; LDH: lactate dehydrogenase; ALT: alanine aminotransferase; ALP: alkaline phosphatase; AST: aspartate aminotransferase.

**Table 6:** Effects of dietary protein level and amounts of rumen-protected lysine and methionine supplementation on the serum antioxidant index of yaks.

Item	Low Protein			High Protein			SEM	*p*-Value		
	CON1	LRPML1	HRPML1	CON2	LRPML2	HRPML2		CP	AA	CP × AA
SOD (U/mL)	466.31	461.19	435.56	424.49	465.46	456.92	7.057	0.711	0.529	0.223
MDA (nmol/mL)	3.41 ^a^	5.94 ^b,c^	6.88 ^c^	4.64 ^a,b^	4.06 ^a^	5.02 ^a,b^	0.344	0.081	0.018	0.028
GSH-Px (U/mL)	36.69 ^a^	62.70 ^b^	60.43 ^b^	38.13 ^a^	42.59 ^a^	41.73 ^a^	2.694	<0.001	<0.001	0.001
CAT (U/mL)	2.28	3	2.26	1.94	2.18	2.17	0.098	0.02	0.057	0.157
T-AOC (U/mL)	2.34	5.12	4.93	1.97	3.86	2.92	0.333	0.007	0.001	0.203

^a–c^ Values within a row lacking a common superscript differ (*p* < 0.05) due to diet protein–rumen-protected methionine and lysine supplementation interactions. CON1: low-protein diet supplemented with no rumen-protected methionine and lysine; LRPML1: low-protein diet supplemented with 0.05% rumen-protected methionine and 0.15% rumen-protected lysine; LRPML2: low-protein diet supplemented with 0.1% rumen-protected methionine and 0.3% rumen-protected lysine; CON2: high-protein diet supplemented with no rumen-protected methionine and lysine; HRPML1: high-protein diet supplemented with 0.05% rumen-protected methionine and 0.15% rumen-protected lysine; HRPML2: high-protein diet supplemented with 0.1% rumen-protected methionine and 0.3% rumen-protected lysine; SOD: superoxide dismutase; MDA: malondialdehyde; GSH-Px: glutathione peroxidase; CAT: catalase; T-AOC: total antioxidant capacity.

**Table 7:** Effects of dietary protein level and amounts of rumen-protected lysine and methionine supplementation on serum-free amino acids of yaks (μg/mL).

Item	Low Protein			High Protein			SEM	*p*-Value		
	CON1	LRPML1	HRPML1	CON2	LRPML2	HRPML2		CP	AA	CP × AA
EAA										
Tryptophan	0.49 ^a^	0.92 ^b^	1.23 ^b^	0.98 ^b^	1.00 ^b^	1.18 ^b^	0.067	0.043	0.003	0.036
Phenylalanine	0.82	1.17	1.2	1.2	1.13	1.24	0.045	0.117	0.107	0.079
Threonine	3.32	3.11	3.06	3.41	3.16	3.5	0.063	0.153	0.329	0.413
Isoleucine	1.14	1.51	1.4	1.41	1.29	1.36	0.041	0.997	0.344	0.058
Leucine	1.46	1.8	1.68	1.58	1.35	1.44	0.053	0.052	0.842	0.057
Valine	2.45 ^a,b^	2.84 ^b^	2.45 ^a,b^	2.59 ^a,b^	2.26 ^a^	2.36 ^a^	0.064	0.11	0.494	0.044
Lysine	0.90 ^a^	1.64 ^c,d^	1.88 ^d^	1.19 ^a,b^	1.33 ^b,c^	1.65 ^c,d^	0.092	0.356	<0.001	0.042
Methionine	0.72 ^a^	1.13 ^a,b^	1.57 ^b,c^	0.72 ^a^	1.18 ^a,b,c^	1.73 ^c^	0.142	0.018	0.06	0.002
NEAA										
Serine	1.47	1.19	1.27	1.12	1.08	1.25	0.044	0.054	0.224	0.205
Glutamic acid	2.44	2.57	2.81	2.76	2.56	2.44	0.052	0.824	0.873	0.167
Glycine	2.68 ^a^	3.34 ^b^	2.90 ^a^	2.87 ^a^	2.50 ^a^	2.52 ^a^	0.086	0.009	0.271	0.009
Alanine	1.56 ^a^	2.42 ^c^	2.21 ^b,c^	1.95 ^b^	2.06 ^b^	2.19 ^b,c^	0.075	0.997	0.001	0.009
Cysteine	0.23 ^a^	0.81 ^b,c,d^	1.06 ^d^	0.63 ^b^	0.75 ^b,c^	1.00 ^c,d^	0.075	0.214	<0.001	0.038
Tyrosine	1.15	1.42	1.33	1.62	1.45	1.52	0.05	0.217	0.825	0.108
Histidine	0.71	1.09	1.15	1.1	1	1.21	0.052	0.168	0.071	0.088
Arginine	1.91 ^a^	3.22 ^b^	3.35 ^b^	3.51 ^b^	3.25 ^b^	3.18 ^b^	0.145	0.001	0.003	<0.001
Proline	0.45 ^a^	0.94 ^b^	1.17 ^b^	0.91 ^b^	0.92 ^b^	1.09 ^b^	0.065	0.141	0.003	0.027
Aspartic acid	0.21	0.38	0.62	0.39	0.39	0.52	0.04	0.612	0.019	0.205

^a–d^ Within a row lacking a common superscript differ (*p* < 0.05) due to diet protein × rumen-protected methionine and lysine supplementation. CON1: low-protein diet supplemented with no rumen-protected methionine and lysine; LRPML1: low-protein diet supplemented with 0.05% rumen-protected methionine and 0.15% rumen-protected lysine; LRPML2: low-protein diet supplemented with 0.1% rumen-protected methionine and 0.3% rumen-protected lysine; CON2: high-protein diet supplemented with no rumen-protected methionine and lysine; HRPML1: high-protein diet supplemented with 0.05% rumen-protected methionine and 0.15% rumen-protected lysine; HRPML2: high-protein diet supplemented with 0.1% rumen-protected methionine and 0.3% rumen-protected lysine; EAA: essential amino acids; NEAA: none-essential amino acids.

## Data Availability

The data presented in this study are available in this article. Further information is available upon request from the corresponding author.
